# On the Influence of the Menthol Moiety on the Transport Properties of a Homologue Series of Functionalized Bis(trifluoromethylsulfonyl)imide Room-Temperature Ionic Liquids: A Quest for the Structure–Property Relationship

**DOI:** 10.1021/acs.jpcb.1c03827

**Published:** 2021-07-23

**Authors:** Joanna Feder-Kubis, Ramesh L. Gardas, Monika Geppert-Rybczyńska

**Affiliations:** † Faculty of Chemistry, Wrocław University of Science and Technology, Wybrzeże Wyspiańskiego 27, Wrocław 50-370, Poland; ‡ Department of Chemistry, Indian Institute of Technology Madras, Chennai, Tamil Nadu 600036, India; § Institute of Chemistry, University of Silesia, Szkolna 9, Katowice 40-006, Poland

## Abstract

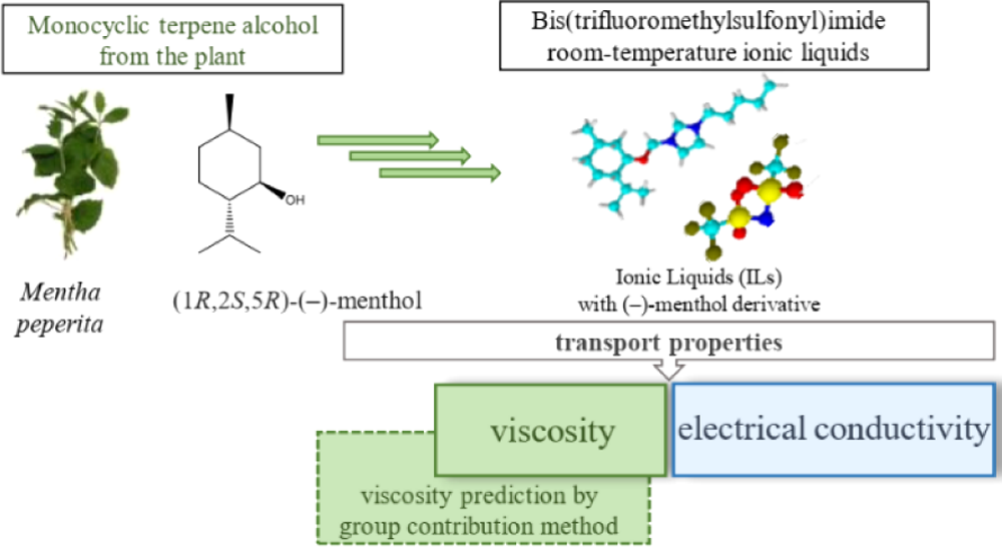

This study explores the transport properties of bis(trifluoromethylsulfonyl)imide-based ionic liquids with a naturally derived (1*R*,2*S*,5*R*)-(−)-menthol moiety in the cationic part. In particular, we investigated the dependence of the dynamic viscosity and electrical conductivity as functions of the alkyl chain length. An important finding of this study is that both properties show nonmonotonic behavior with respect to the alkyl chain length. The nonmonotonic dependency is an obstacle for establishing the relationships between the structure and transport properties of homologues. To overcome this difficulty, we recommend fast property screening using a theoretical model that we developed, which allows for efficient viscosity prediction by means of the group contribution method. As demonstrated in this study, the model allows for reliable predictions of viscosity in the studied series with an overall relative deviation of less than 8%.

## Introduction

Ionic liquids (ILs) are organic salts that melt at temperatures below 100 °C, which is an arbitrary temperature point that is frequently used as part of the definition of this class of compound. Room-temperature ILs (RTILs), in particular, occupy a privileged spot as they are liquid near 25 °C. Due to this feature, they have remained in the limelight for the last two decades.^1,2^ In 1992, landmark work by Wilkes and Zaworotko^3^ described “air- and water-stable ILs” and thus sparked a plethora of papers devoted to these compounds. Nowadays, the most investigated ILs or RTILs are salts that are synthesized for special applications due to their specific properties.^4−12^ The versatile applications of ILs can be linked with the ease of their functionalization, which is a route for controlling the structure of ILs and their corresponding physicochemical properties.

The palette of IL applications is constantly expanding due to its desirable physicochemical properties, which include extremely low vapor pressure at ambient temperature, high thermal stability, wide liquid range, tunable viscosity, and high conductivity.^4−12^ Transport properties, such as the viscosity and ionic conductivity, are among the most relevant attributes for chemical process design and development.^13^ The relationships between specific properties and the structure of ILs are a key factor that is discussed extensively in the literature. Various structural elements are considered, including the dependency of transport properties on the length of the alkyl chain, anions, the type of basic core, and the presence of special functional groups.^14−18^ These are all examined for better recognition of the dependencies that have a direct impact on the selection of ILs with properties tailored for target applications. Particularly noteworthy are the dependencies that are not regular, in which case, the predictability of the properties and the design of the desired compounds are much more limited.

In the past, ILs have undoubtedly been regarded as eco-friendly alternatives to classical organic solvents in many applications.^1,2,6^ The replacement of common fossil-fuel-based organic solvents with green counterparts with several desired features seems to be an important step in the development of green and clean chemical technologies. Such features include low vapor pressure even at high temperatures, low flammability, and little or no toxicity.^19^ ILs occupy a particular place among several environmentally benign reaction media (such as water, supercritical fluids, fluorous solvents, and alcohols), and their appreciated features include extremely low vapor pressure, good solvating properties, reasonable thermal stability, and easily tunable chemical properties (polarity, acidity, and basicity) and physical properties (e.g., viscosity). However, it turns out that they might have a negative impact on the environment.^20,21^ This has motivated the efforts of many scientists including our group to synthesize ILs with moieties that are partially or entirely derived from natural components (bio- or biomass-based ILs).^22−24^

This requirement is fulfilled by imidazolium bis(trifluoromethylsulfonyl)imides with a naturally derived (1*R*,2*S*,5*R*)-(−)-menthol moiety in the cationic part, which we have synthesized previously.^25,26^ Our first work on this group of ionic compounds with a natural terpene substituent^25^ initiated a very interesting series of subsequent papers that clarified obtaining particularly pure ILs with a wide range of applications.^26−29^ Both the process of obtaining these ionic compounds and the selection of the special raw substituent (cheap, commercially available, and widely used monoterpene alcohol) are beneficial for sustainable development and can be regarded as an innovative alternative to typical solvents. A wide set of physicochemical parameters of these compounds has been thoroughly studied, including the decomposition temperature, glass temperature, specific rotation, refractive index, density, kinematic viscosity, speed of sound, isobaric heat capacity, surface tension, and contact angles on certain solid materials.^25−27^ These measurements have often been performed in a wide temperature range at atmospheric pressure (e.g., *p* = 0.1 MPa). It has been demonstrated that most of these properties have very irregular behavior as a function of the number of methylene groups, which is in agreement with our work and the literature.^25−27,30,31^ ILs with the (−)-menthol substituent have also been tested by our group for their catalytic activity (cycloisomerization and Diels–Alder reaction).^28,29^ Another beneficial feature of these salts is their high antielectrostatic activity.^25^ For such applications, the method of applying a substance that removes the electric charge from a polymer surface is crucial from a technical perspective and is directly related to the viscosity of the applied system.

To better assess the potential of ILs for such applications, it is mandatory to thoroughly investigate their transport properties. The viscosity (η) of a solvent is an essential factor for stirring, diffusion, mass transfer, and other processes and could have a significant influence on the cost and efficiency. This is the rationale behind the need for our recent studies on the transport properties (including the viscosity) of renewable-based solvents with numerous additional functional features. Given the potential applications of [C_*n*_-im-CH_2_OMen][NTf_2_] ILs [*n* = 1–10], we extended our previous efforts and experimentally studied the transport properties of this homologue series, particularly viscosity and conductivity. One important part of this work is the assessment of a theoretical model that allows for efficient viscosity reproduction by means of the group contribution method.^32,33^

## Materials and Methods

### Materials

RTILs with a natural monoterpene derivative, 3-alkyl-1-[(1*R*,2*S*,5*R*)-(−)menthoxymethyl]imidazolium bis(trifluoromethylsulfonyl)imides ([C_*n*_-im-CH_2_OMen][NTf_2_], *n* = 1–10), were synthesized with satisfactory yield (higher than 96%).^26^ The impurity levels found by ion chromatography (IC) were extremely low and sometimes even below the detection limit (Table 1). Table 1 presents the abbreviations used, glass transition temperature *T*_g_ (K), density ρ (g·cm^–3^) at 298.15 K, and the water content in investigated RTILs.^27^

**Table 1:**
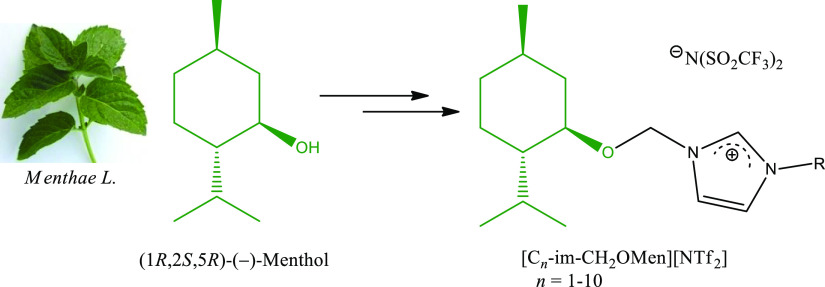
Properties of 3-Alkyl-1-[(1*R*,2*S*,5*R*)-(−)-Menthoxymethyl]imidazolium Bis(trifluoromethylsulfonyl)imides, [C_*n*_-im-CH_2_OMen][NTf_2_] ILs with *n* = 1–10: Final Mass Fraction Purity, Onset Temperature of Thermal Decomposition *T*_onset_ (K), Glass Transition Temperature *T*_g_ (K), Density ρ (g·cm^–3^) at 298.15 K, and Final Mass Fraction Water Content from Coulometric Carl-Fisher Titration^27^

IL [C_*n*_-im-CH_2_OMen][NTf_2_]	R	final mass fraction purity^a^	*T*_onset_ (K)^b^	*T*_g_ (K)^b^	ρ (g·cm^–3^)^c^	*w*_H_2_O_·10^4^^c^
[C_1_-im-CH_2_OMen][NTf_2_]	CH_3_	0.9996	503.15	229.15	1.3320	1.90
[C_2_-im-CH_2_OMen][NTf_2_]	C_2_H_5_	0.9998	498.15	227.15	1.3104	1.13
[C_3_-im-CH_2_OMen][NTf_2_]	C_3_H_7_	0.9998	503.15	225.15	1.2875	1.20
[C_4_-im-CH_2_OMen][NTf_2_]	C_4_H_9_	0.9998	503.15	222.15	1.2733	1.04
[C_5_-im-CH_2_OMen][NTf_2_]	C_5_H_11_	0.9996	503.15	223.15	1.2570	0.76
[C_6_-im-CH_2_OMen][NTf_2_]	C_6_H_13_	0.9998	498.15	223.15	1.2350	1.38
[C_7_-im-CH_2_OMen][NTf_2_]	C_7_H_15_	0.9998	503.15	222.15	1.2241	0.51
[C_8_-im-CH_2_OMen][NTf_2_]	C_8_H_17_	0.9998	498.15	223.15	1.2152	0.97
[C_9_-im-CH_2_OMen][NTf_2_]	C_9_H_19_	0.9992	503.15	222.15	1.2008	0.11
[C_10_-im-CH_2_OMen][NTf_2_]	C_10_H_21_	0.9999	503.15	222.15	1.1889	0.27

a Values taken form ref (26) (the same synthetic portion as for viscosity and conductivity measurement).

b Values taken form ref (25).

c Values taken from ref (27) (the same synthetic portion after drying as for viscosity and conductivity measurement).

## Methods

The dynamic viscosity η (mPa·s) of some 3-alkyl-1-[(1*R*,2*S*,5*R*)-(−)-menthoxymethyl]imidazolium bis(trifluoromethylsulfonyl)imide homologues (*n* = 3, 4, 6, 9, and 10) was obtained from the kinematic viscosity ν (mm^2^·s^–1^), which was measured with a micro-Ubbelhode viscometer (SI Analytics, capillaries IIc and III) based on the relation η = ν·ρ, where ρ is the density.^27^ For the homologues [C_*n*_-im-CH_2_OMen][NTf_2_] with *n* = 1, 2, 5, 7, and 8, the dynamic viscosity was obtained directly from a microviscometer (Lovis 2000 ME), which was connected to an Anton Paar DSA 5000M apparatus. The temperature range for all measurements was 298.15–328.15 K with a step size of 5 K.

The micro-Ubbelohde viscometer was certified with a certificate of calibration from the manufacturer in accordance with DIN 55 350, part 18. For these measurements, about 4–5 mL of the sample was used, and the measurements were repeated 5–10 times. If necessary, a time correction for the viscous flow was applied. The estimated precision of the viscosity measurements was ±0.3%. The temperature was measured with Pt—100 Ω with a resolution of 0.01 K and an uncertainty of ±0.05 K. A rolling ball microviscometer (Lovis 2000 ME) with a 2.5 mm capillary was used. The temperature was controlled within ±0.02 K. The viscosity repeatability and accuracy reported by the manufacturer are 0.1 and 0.5%, respectively. This apparatus enables measurements in a wider temperature range than the one presented in this work, and in order to unify all calculations, we decided to use values determined in the temperature range of 298.15–328.15 K.

The specific conductivity κ (mS·cm^–1^) was measured with a conductivity meter (Elmetron CC—511), which was equipped with a Hydromet CDM—2 electrode with a cell constant *k* = (0.63 ± 0.01) cm^–1^. The cell was calibrated with a standard aqueous KCl solution. The accuracy of the measurements is in accordance with the producer’s estimation of ±0.5%. The temperature values were read from a platinum thermometer placed in the measuring cell with a resolution of 0.01 K and an uncertainty of ±0.05 K.

## Results

### Experimental Results

**Figure 1:**
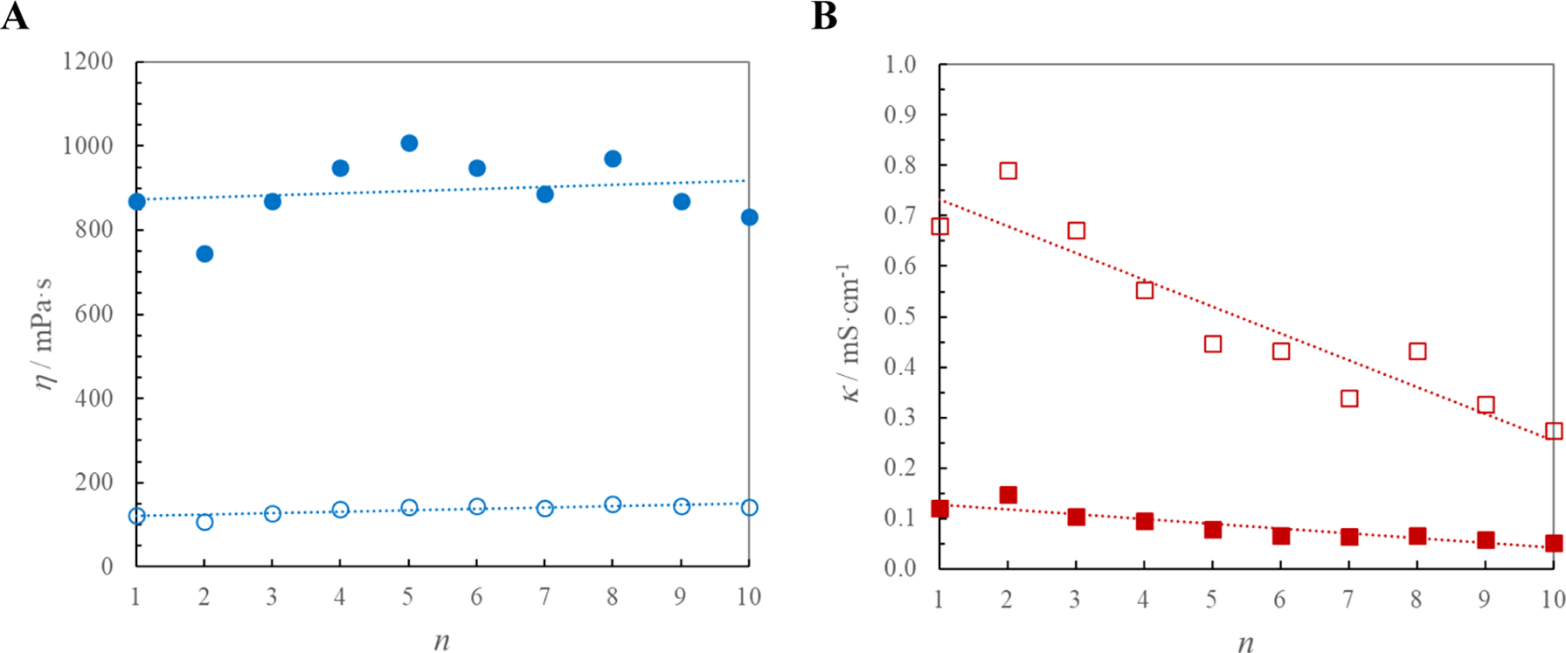
Dynamic viscosity (A) and conductivity (B) of [C_*n*_-im-CH_2_OMen][NTf_2_] ILs *vs n* (*n* = 1–10) at *T* = 298.15 K (filled circles or squares) and at *T* = 323.15 K (empty circles or squares); lines are guide to eye to observe the trend.

Tables S1 and S2 in Supporting Information present the kinematic viscosity of 3-alkyl-1-[(1*R*,2*S*,5*R*)-(−)-menthoxymethyl]imidazolium bis(trifluoromethylsulfonyl)imide homologues (*n* = 3, 4, 6, 9, and 10) and dynamic viscosity of all homologues. Figure S1 in Supporting Information presents the dynamic viscosity of the C_*n*_-im-CH_2_OMen][NTf_2_] ILs, and Figure 1a shows the dependency of η on the length of alkyl chain in the (−)-menthoxymethylimidazolium cation at *T* = 298.15 K and 323.15 K. The rounded kinematic viscosity at 303.15 K for 3-alkyl-1-[(1*R*,2*S*,5*R*)-(−)-menthoxymethyl]imidazolium bis(trifluoromethylsulfonyl)imides has been published previously.^25^ Thus, a comparison of the previous results with those obtained in this study at only one temperature *T* = 303.15 K is presented in Figure S2 in Supporting Information.

The most important element influencing the differences of the obtained kinematic viscosity values is related to the use of different purification protocols applied in the current and previous work. In our previous paper on (−)-menthol-based bis(trifluoromethylsulfonyl)imides,^25^ the discussed ILs were only washed with distilled water. The water content was then given generally as less than 500 ppm. The level of the ionic impurities was not studied in detail, and only their absence was confirmed by the method based on AgNO_3_. Over time, we have improved our research facilities, and we carried out the purification of ILs following a procedure involving several steps (extraction several times with distilled water, dissolution in acetone, placing acetone-IL solutions in the fridge, etc.).^26^ Currently, we analyze the impurities in great detail using IC analysis. Thus, it is difficult to compare the level of salt impurities for the ILs tested in the present work (which are known in detail) with those presented in our first work.^25^ Furthermore, in that study,^25^ the kinematic viscosity was measured with a micro-Ostwald viscometer with no information on uncertainty.

Table S3 in Supporting Information presents the specific conductivity of 3-alkyl-1-[(1*R*,2*S*,5*R*)-(−)-menthoxymethyl]imidazolium bis(trifluoromethylsulfonyl)imides. Figure S3 in Supporting Information shows the conductivity of [C_*n*_-im-CH_2_OMen][NTf_2_] ILs at different temperatures. Figure 1b shows the dependency of κ on the length of the alkyl chain in the menthoxymethylimidazolium cation at *T* = 298.15 and 323.15 K.

The general trend of the viscosity (see Figures S1 and 1a) falls within expectations: the smallest value of η is observed for the short alkyl substituent in the bis(trifluoromethylsulfonyl)imides, and its elongation is accompanied by an increase in viscosity. However, this dependency is not as regular as anticipated based on the results for the most often studied 1-alkyl-3-methylimidazolium ILs with tetrafluoroborate, BF_4_^–^, hexafluorophospate, PF_6_^–^, or even bis(trifluoromethylsulfonyl)imide, NTf_2_^–^ anions (see Figure S4 in Supporting Information).^14,34−45^ This irregularity can be explained by the relatively small variation of the quantity in question *versus n*. Furthermore, irregularity has been observed previously for this class of compounds in the case properties such as the speed of sound, refractive index (including nonmonotonic behavior for first homologues),^26^ surface tension,^27^ and glass temperature.^25^ Previous reports have indicated a small influence of the length of the alkyl chain in the cation (with nonmonotonic behavior) on certain transport properties of ILs with a bis(trifluoromethylsulfonyl)imide anion.^46,47^

The electrical conductivity changes more regularly,^48,49^ or else, an odd-even effect takes place for 1-alkyl-1-methylmorpholinium dicyanamide ILs [C_*n*_C_1_mo][DCA]^50^ (see Figure S5 in Supporting Information). Compared to the viscosity, the conductivity of 3-alkyl-1-[(1*R*,2*S*,5*R*)-(−)-menthoxymethyl]imidazolium bis(trifluoromethylsulfonyl)imides changes more significantly with the elongation of the alkyl chain in the cation (see Figure S3 in Supporting Information and Figure 1b). This quantity varies to a larger extent for all homologues of other classes of previously examined compounds with the same cation 1-alkyl-3-methylimidazolium and different anions of tetrafluoroborate, BF_4_^–^, hexafluorophosphate, PF_6_^–^, or even bis(trifluoromethylsulfonyl)imide, NTf_2_^–^, as shown in Figure S6 in Supporting Information(40,51−57) and in a previous study.^46^

Due to their nonlinear behavior, the temperature dependencies of the viscosity and conductivity can be described with the empirical Vogel–Fulcher–Tammann (VFT) equations^46,55,58−60^
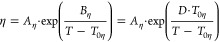
1
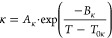
2where *A*_η_ and *A*_κ_ are the limiting viscosity and conductivity, respectively; *B*_η_ and *B*_κ_ denote fitting parameters, and *T*_0η_ and *T*_0κ_ are the ideal glass transition temperatures. The parameter *D* presented on the right-hand side of VFT eq 1 is related to the strength/fragility of the substance that controls how closely the substance obeys the Arrhenius law (*D* = ∞).^61^ All parameters in eqs 1 and 2 are presented in Tables S4 and S5 in Supporting Information.

As *T*_0η_ and *T*_0κ_ values for the viscosity and conductivity (eqs 1 and 2), a common value of 165.06 K was applied, which is lower than the experimental *T*_g_ (see Table 1 in Materials and Methods). This was an optimum value taken from calculations for viscosity prediction by the group contribution method (the details will be discussed).

The strength parameter *D* is near 10 (see Table S4 in Supporting Information) for the most fragile glass-forming materials, which reveals the largest deviations from the Arrhenius law.^62^ According to Böhmer et al.,^62^ such fragile glass formers are substances with nondirectional interatomic/intermolecular bonds, such as molten salts or ILs.^63^ All *D* values obtained in this work are only a bit higher than those observed for 1-alkyl-3-methylimidazolium or 1-alkyl-1-methylpirrolidinium bis(trifluoromethylsulfonyl)imide ILs.^46,63^ The relationship between the conductivity and viscosity of ILs is expressed by the fractional Walden rule^55,60^
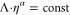
3where Λ is the molar conductivity and α is an index determined from the slope of the line log Λ(log 1/η). The values of Λ can be calculated using the following equation
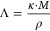
4where *M* is the molecular mass of the IL. In this work, the molar conductivity in the temperature range of 293–323 K calculated from the electrical conductivity (Table S3 in Supporting Information), molecular mass (from Table 1 in Materials and Methods), and the density taken from earlier work (measured for the same sample as conductivity)^27^ are given in Table S6 in Supporting Information.

**Figure 2:**
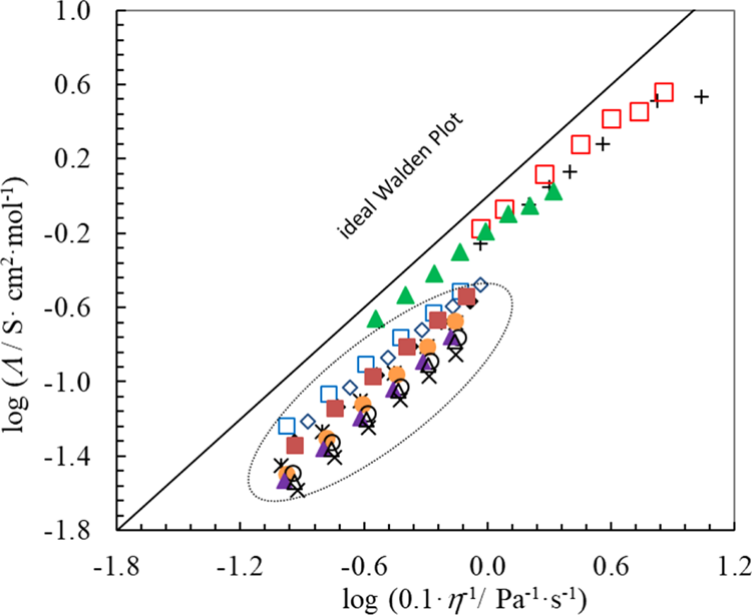
Walden plot for 3-alkyl-1-[(1*R*,2*S*,5*R*)-(−)-menthoxymethyl]imidazolium bis(trifluoromethylsulfonyl)imides—points in an oval: filled diamonds—C_1_; empty diamonds—C_2_; filled squares—C_3_; empty squares—C_4_; stars—C_5_; filled circle—C_6_; empty circle—C_7_; filled triangle—C_8_; empty triangle—C_9_; and multiplication sign—C_10_ and for chosen representatives of other series of ILs: pluses—[C_4_C_1_im][NTf_2_],^40^ empty squares—[C_4_C_1_im][BF_4_],^60^ and filled triangles—[C_4_C_1_im][PF_6_].^66^ The solid line represents the ideal Walden line for diluted KCl aqueous solutions.^65^

Figure 2 shows the dependencies of the molar electrical conductivity Λ on the fluidity 1/η in logarithmic coordinates, as well as the ideal line (with a slope of α = 1) for a dilute KCl solution in a fully dissociated system of ions of equal mobility.^64,65^ However, some literature reports show that for infinitely diluted KCl solutions, α is not 1 but 0.87.^55^ For the investigated 3-alkyl-1-[(1*R*,2*S*,5*R*)-(−)-menthoxymethyl]imidazolium bis(trifluoromethylsulfonyl)imide ILs, the log Λ = log (1/η) lines lie below the ideal one, and α is between 0.86 and 1 (see Table S7 in Supporting Information).

The solid line for diluted KCl solution in Figure 2 was assumed to be the reference line despite the fact that its theoretical meaning has no importance for comparison to the ILs; it is assumed to be a good calibration point.^55,60,67,68^

### Prediction of Viscosity Based on the Group Contribution Method

The experimental viscosity data were fitted with the logarithmic form of the VFT equation (eq 1) (ln η = ln *A*_η_ + *B*_η_/(*T* – *T*_0η_)). It was successfully used to model the temperature dependency of the viscosity of an IL^33^ and applied to the data in Table S2 in Supporting Information. The fit is presented in Figure S1. *A*_η_ and *B*_η_ in eq 1 can be obtained by a group contribution method according to eq 5
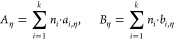
5where *n*_*i*_ is the number of groups of type *i*, *k* is the total number of different groups in the molecule, and the parameters *a*_*i*,η_ and *b*_*i*,η_ are estimated and presented in Table 2 (group parameters *a*_*i*,η_ and *b*_*i*,η_).

**Table 2:** Group Parameters *a*_*i*,η_, and *b*_*i*,η_ Taken from ref (33) or Obtained in This Work

group parameters *a*_*i*,η_				group parameters *b*_*i*,η_			
C_1_im	CH_2_OMen	[NTf_2_]	CH_2_	C_1_im	CH_2_OMen	[NTf_2_]	CH_2_
–7.271	3.036	–1.119	0.007528	510.51	999.03	94.2	0.4092

In this work, we obtained parameters *a*_*i*,η_ and *b*_*i*,η_ for the menthoxymethyl group (CH_2_OMen) and the *b*_*i*,η_ parameter of the methylene group (CH_2_). Other group parameters were taken from earlier work.^33^ They were calculated based on the viscosity correlation for ILs with different cations and anions in a wide range of temperature.

The VTF equation was fitted to the experimental viscosity data, which comprised 70 data points in total for 10 studied ILs and covered wide ranges of temperature (298.15–328.15 K) and viscosity (82–1009 mPa·s). It was found that *T*_0η_ was almost constant for all the ILs with a value close to 165 K. A simultaneous optimization of the entire database was performed using the objective function (O.F.) described in eq 6. The result showed that the optimum value of *T*_0η_ is 165.06 K, which is similar to the value proposed for various classes of ILs.^33^
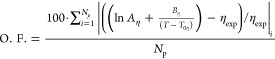
6

The relative average deviation (RD) is defined as
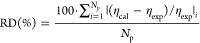
7where subscripts “cal” and “exp” denote calculated and experimental properties, respectively, and *N*_p_ is the number of available data points for each system reported in Table S8 in Supporting Information (RD values were obtained from eq 7 for each IL).

**Figure 3:**
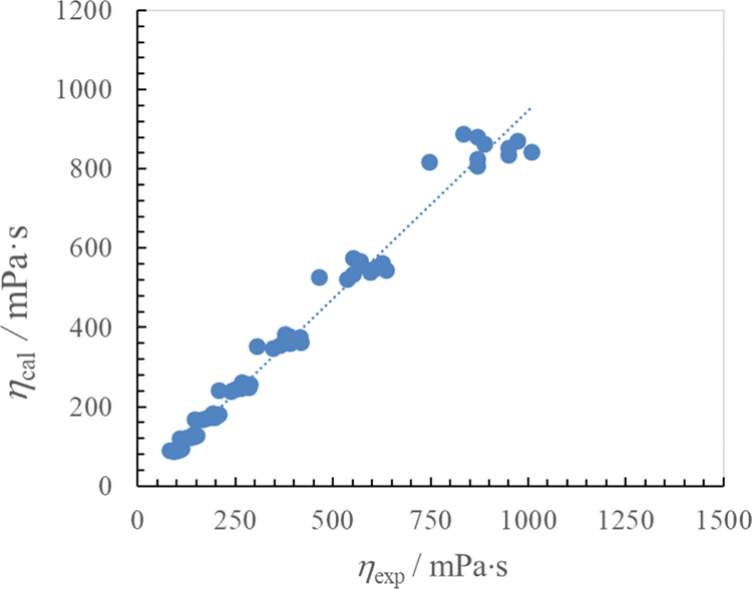
Linear relationship between experimental and calculated viscosity using the logarithmic form of eq 1 for 3-alkyl-1-[(1*R*,2*S*,5*R*)-(−)-menthoxymethyl]imidazolium bis(trifluoromethylsulfonyl)imide ILs.

As shown in Figure 3, there is very good agreement between the calculated and experimental viscosity data obtained from the VTF equation (eqn 1) with *T*_0η_ = 165.06 K and group contribution parameters *a*_*i*,η_ and *b*_*i*,η_ (Table 2).

## Discussion

In the present work, the additional impact on viscosity behavior of homologue series of [C_*n*_-im-CH_2_OMen][NTf_2_], *n* = 1–10, is presumably caused by the presence of the 3-alkyl-1-[(1*R*,2*S*,5*R*)-(−)-menthoxymethyl]imidazolium cation, which is an interesting alternative to 1-alkyl-3-methylimidazolium. However, for the symmetrical 1,3-dialkylimide bis(trifluoromethylsulfonyl)imide ILs, the dependency of η(*n*) is also very small (see Figure S4 in Supporting Information). What is more, for all bis(trifluoromethylsulfonyl)imide ILs, a minimum for η(*n*) dependence and a maximum for κ(*n*) are present for C_2_ homologues (Figures S4 and S6 in Supporting Information). This situation is common for viscosities of ILs taken from the literature and those obtained in the presented work.

In all ILs, the local nanostructure is related to Coulombic interactions between ions, which cause a more isotropic distribution of ionic species and the van der Waals and hydrogen bonding, providing an anisotropic distribution.^69^ The increasing alkyl chain length supports larger, more distinct, apolar domains, as a concurrence with the electrostatic interactions between charged sites creating polar domains. In this way, the IL nanostructure (visible polar and apolar domains) occurs. The relative dimensions of the polar and nonpolar moieties of cations and anions influence a packing geometry (alkyl apolar/polar) and control the preferred arrangement of polar and nonpolar domains.^69,70^ The presence of the (1*R*,2*S*,5*R*)-(−)-menthol moiety in the imidazolium cation may affect the IL nanostructure as the steric hindrance, not only due to the geometry but also due to the rotational dynamics of the substituents in the cationic families, what can determine the strength of the interaction between the cation and the anion.^69^ Presumably, it also hinders the development of apolar domains, which is demonstrated in slight change for some physicochemical properties *versus* the alkyl chain length. It seems that this effect should be more pronounced in the case of transport properties as viscosity and the speed of sound and conductivity for longer chain homologues. These properties change distinctly only for the first few homologues, and from [C_5_-im-CH_2_OMen][NTf_2_] or [C_6_-im-CH_2_OMen][NTf_2_], the variation of viscosity, speed of sound, conductivity, and others, *T*_g_ and surface tension, is only slight and often unclear (see also refs (25)–^27^). It is worthy of notice that in the literature, the existence of the ionic pairs and free volume effect is also regarded, when the relation between the transport properties and IL structure is discussed.^69^ However, we believe that our simplest approach can be the most reasonable due to the following premises.

The literature indicates that there are few IL classes for which some irregular behavior was found for the viscosity *versus* the length of the alkyl chain in the imidazolium cation. Some of the data have been obtained from one source, such as 1-alkylpyridinium bis(trifluoromethanesulfonyl)imides, [C_*n*_py][NTf_2_],^71^ 1-alkyl-1-methylpiperridinium bis(trifluoromethanesulfonyl)imides, [C_*n*_C_1_pip][NTf_2_],^48^ 1-alkylthiolanium bis(trifluoromethanesulfonyl)imides, [C_*n*_tl][NTf_2_],^49^ 1-alkyl-1-methylmorpholinium dicyanamides, [C_*n*_C_1_mo][DCA],^50^ and 1-alkyl-4-methyltriazolium bis(trifluoromethanesulfonyl)imides, [C_*n*_C_1_-4-tz][NTf_2_].^72^ In some cases, as for 1-alkyl-1-methylpyrrolidinium dicyanamides, [C_*n*_C_1_pyr][DCA],^42,73,74^ and 1-alkyl-3-methylimidazolium trifluoromethanesulfonates, [C_*n*_C_1_im][TFO],^34,75−77^ this irregularity may be a consequence of different origins, profiles of impurities, and measurement methods in different studies. Figure S7 in Supporting Information shows the viscosity for an example series of ILs with irregular viscosity behavior in the homologue series.

The Walden rule is interpreted similarly to the Stokes–Einstein relation between the self-diffusivity *D*_*i*_ of species *i* in a medium of viscosity η and hydrodynamic radius *r*_*i*_ (*D*_*i*_ = *k*_b_*T*/6πη*r*_*i*_, where *k*_b_ is the Boltzmann constant and *T* is the temperature). In this case, *k*_b_*T* represents the thermal energy required to overcome the viscous force of the medium during particle flow (possibly the frictional force, which impedes particle movement). Surprisingly, the Stokes–Einstein relation can be successfully applied for not only solutions (where large ions move in a solvent composed of small molecules) but also for pure ILs.^64^ Furthermore, the deviation from the Walden rule is usually interpreted in terms of decreasing ionicity by association.^67^ 3-Alkyl-1-[(1*R*,2*S*,5*R*)-(−)-menthoxymethyl]imidazolium bis(trifluoromethylsulfonyl)imides consist of large ions, so their surface charge density is relatively low. Both ions (especially the cation) have a very complex structure, including groups and atoms that can be involved in some specific and nonspecific interactions. The size of ions, their complex structure, and possible interactions can limit ion mobility, which is why the deviation from the Walden lines in Figure 3 for all [C_*n*_-im-CH_2_OMen][NTf_2_] ILs comes as no surprise. For the sake of comparison, Figure 3 also includes Walden plots for three representatives of the most well-known groups of ILs: [C_4_C_1_im][NTf_2_], [C_4_C_1_im][BF_4_], and [C_4_C_1_im][PF_6_].^40,55,60^

Apparently, all α parameters for [C_*n*_-im-CH_2_OMen][NTf_2_] (see Table S7 in Supporting Information) have typical values like other groups of ILs,^46,55,60^ but the distance of their Walden plots from the “ideal line” is more substantial. This implies that the relation between the conductivity and viscosity of the homologue series is similar to that of other IL groups. However, the ionicity of the investigated imides with a methyl group in the alkyl chain (in Table S7 in Supporting Information), between 0.2 and 0.4, is much lower than that observed for other ILs. To sum up, all 3-alkyl-1-[(1*R*,2*S*,5*R*)-(−)-menthoxymethyl]imidazolium bis(trifluoromethylsulfonyl)imides can be regarded as typical or good ILs despite their large viscosity along with low conductivity.^46,64^

**Figure 4:**
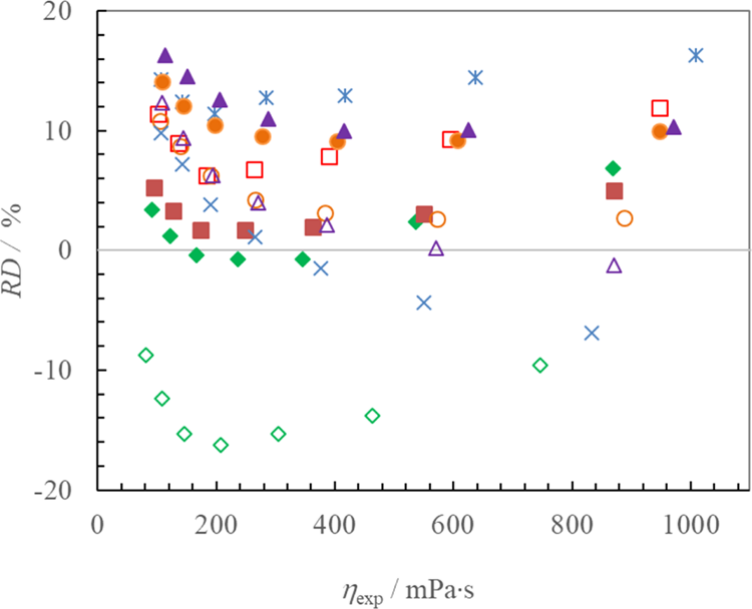
Relative deviations between that calculated using eq 7 and experimental viscosity data as a function of experimental viscosity for 3-alkyl-1-[(1*R*,2*S*,5*R*)-(−)-menthoxymethyl]imidazolium bis(trifluoromethylsulfonyl)imide ILs in the current study; points: filled diamonds—C_1_; empty diamonds—C_2_; filled squares—C_3_; empty squares—C_4_; stars—C_5_; filled circle—C_6_; empty circle—C_7_; filled triangle—C_8_; empty triangle—C_9_; and multiplication sign—C_10_.

The calculated viscosity (η_cal_) of the ILs shows good agreement with the corresponding experimental viscosity (η_exp_), where ln(η_cal_) = (0.947 ± 0.009)·ln(η_exp_) (*R*^2^ = 0.983 at the 95% confidence level). Figure 4 shows the relative deviations between the calculated and experimental viscosity data as a function of the experimental viscosity for all data points used in the current study. For the 70 data points of the 10 studied ILs, the overall RD was 7.9% with a maximum deviation less than 16.3%. Furthermore, 34% of the estimated viscosities were within a relative deviation of 0.0–5.0%, 30% of them were within 5.01–10.0%, 29% of them were within 10.01–15.0%, and only 7% of them had more than 15.0% deviation. The maximum relative deviation was 16.33%, which was observed for [C_8_-im-CH_2_OMen][NTf_2_] and (3-octyl-1-[(1*R*,2*S*,5*R*)-(−)-menthoxymethyl]imidazolium bis(trifluoromethylsulfonyl)imides) at 328.15 K. It seems that there is no trend along the homologue series in the relative deviations for the investigated 3-alkyl-1-[(1*R*,2*S*,5*R*)-(−)-menthoxymethyl]imidazolium bis(trifluoromethylsulfonyl)imides (see Figures 3 and 4).

## Conclusions

In many instances, the functionalized bis(trifluoromethylsulfonyl)imides studied in this work comply with the rules of sustainable development, including energy-saving processes and the use of natural components for synthesis. At the same time, they are very promising from a technological perspective regarding renewable-based solvents, catalysts, or even antielectrostatic agents. Thus, it is of pivotal importance to examine their transport properties to select application-suitable ILs from the homologue series. Motivated by the wide palette of applications of ILs, we investigated the dependency of the dynamic viscosity and electrical conductivity on the alkyl chain length.

The dynamic viscosity and electrical conductivity of 3-alkyl-1-[(1*R*,2*S*,5*R*)-(−)-menthoxymethyl]imidazolium bis(trifluoromethylsulfonyl)imide showed nonmonotonic behavior with respect to the alkyl chain length. This is supposedly the common feature for transport properties of bis(trifluoromethylsulfonyl)imide ILs. Despite this, for the ILs investigated in this work, a minimum for viscosity and maximum for electric conductivity in the η(*n*) and κ(*n*) dependencies for the C_2_ homologue are visible. It seems that there is no trend for relative deviations between those calculated using eq 7 and the experimental viscosity data in connection with the experimental method when the largest RDs (for C_2_, C_5_, and C_8_) were obtained for viscosities from the same experimental technique.

Although such an irregular behavior does not allow for establishing structure–property relationships, one may still greatly benefit from the results obtained in the present work. Namely, in the case of antielectrostatic activity, practically all of the discussed salts show equally good properties of electric discharge from a given surface. Thus, the choice of an appropriate antistatic agent can be solely based on its transport properties (viscosity) in relation to technical and practical use, along with economic considerations.

## Supporting Information Available

Tables listing experimental and calculated kinematic and dynamic viscosity, parameters of VFT equations for these quantities, molar conductivity, linear fitting parameters for fractional Walden plot, ionicity, and relative deviations (RD) between calculated and experimental viscosity and figures showing dynamic viscosity (experimental and calculated), comparison of kinematic viscosity with results from earlier work, experimental conductivity, literature viscosity, and conductivity of other homologues series of ILs with regular and irregular behavior (PDF)
