# Supplementary material for: On the Influence of the Menthol Moiety on the Transport Properties of a Homologue Series of Functionalized Bis(trifluoromethylsulfonyl)imide Room-Temperature Ionic Liquids: A Quest for the Structure–Property Relationship
Source: J Phys Chem B. 2021 Jul 23;125(30):8502–10. doi: 10.1021/acs.jpcb.1c03827 (PMC8389901; doi:10.1021/acs.jpcb.1c03827)
Supplement: Supplementary file 1 — jp1c03827_si_001.pdf [file jp1c03827_si_001.pdf]

Supporting Information

On the Influence of the Menthol Moiety on the Transport Properties of a Homologue Series of Functionalized Bis(trifluoromethylsulfonyl)imide Room-Temperature Ionic Liquids: A Quest for the Structure-Property Relationship

Joanna Feder-Kubis[a], Ramesh L. Gardas[b], Monika Geppert-Rybczyńska[c*]

[a] Faculty of Chemistry, Wrocław University of Science and Technology, Wybrzeże Wyspiańskiego 27, 50-370 Wrocław, Poland

[b] Department of Chemistry, Indian Institute of Technology Madras, Chennai, T.N. 600036, India

[c] Institute of Chemistry, University of Silesia, Szkolna 9, 40-006 Katowice, Poland

[*] to whom correspondence should be addressed: monika.geppert-rybczynska@us.edu.pl

Table S1. Kinematic viscosity, ν ($mm^2 \cdot s^{-1}$) of 1-alkyl-3-[(1*R*,2*S*,5*R*)-(–)-menthoxymetyl]-imidazolium bis(trifluoromethylsulfonyl)imide ionic liquids, [C_n-im-CH_2OMen][NTf_2] ILs (n = 3,4,6,9,10) at T = (298.15–328.15) K and p = 0.1 MPa measured with Micro-Ubbelohde viscometers, capillaries IIc and III.

Table S2. Dynamic viscosity, η (mPa·s) of 1-alkyl-3-[(1*R*,2*S*,5*R*)-(–)-menthoxymetyl]-imidazolium bis(trifluoromethylsulfonyl)imide ionic liquids, [C_n-im-CH_2OMen][NTf_2] ILs (n = 1–10) at T = (298.15–328.15) K and p = 0.1 MPa.

Table S3. Conductivity, κ ($mS \cdot cm^{-1}$) of 1-alkyl-3-[(1*R*,2*S*,5*R*)-(–)-menthoxymetyl]-imidazolium bis(trifluoromethylsulfonyl)imide ionic liquids, [C_n-im-CH_2OMen][NTf_2] ILs (n = 1–10) at T = (293.15–323.15) K and p = 0.1 MPa.

Table S4. Parameters of eq 1 for viscosity (in the manuscript): B_η parameter, limiting viscosity, A_η, Angell's strength parameter, D, with the ideal glass transition temperature, $T_{0\eta}$ = 165.06 K (from the group contribution model).

Table S5. Parameters of eq 2 for conductivity (in the manuscript): B_κ parameter, limiting conductivity, A_κ, with the ideal glass transition temperature, $T_{0\kappa}$ = 165.06 K (from the group contribution model).

Table S6. Molar conductivity, Λ ($S \cdot cm^2 \cdot mol^{-1}$) of 1-alkyl-3-[(1*R*,2*S*,5*R*)-(–)-menthoxymetyl]-imidazolium bis(trifluoromethylsulfonyl)imide ionic liquids, [C_n-im-CH_2OMen][NTf_2] ILs (n = 1–10) at T = (298.15–323.15) K and p = 0.1 MPa.

Table S7. Linear fitting parameters for fractional Walden plots according to linear form of eq 3 (in the manuscript).

Table S8. *RD* values obtained from eq 7 (in the manuscript) for each IL in temperature range T = 298.15 K-328.15 K.

Figure S1. Dynamic viscosity, η, (left) and log η (right) of 3-alkyl-1-[(1*R*,2*S*,5*R*)-(–)-menthoxymethyl]imidazolium bis(trifluoromethylsulfonyl)imides at different temperatures (for better visibility only chosen lines are drawn according to eq 1 in the manuscript, or are straight lines for logarithmic scale).

Figure S2. Kinematic viscosity of [C_n-im-CH_2OMen][NTf_2] ILs *vs. n* (n = 1–10) at T = 303.15 K obtained in this work (squares) in comparison with literature values (diamonds) (Ref. 25)

Figure S3. Conductivity, κ, (left) and log κ (right) of 3-alkyl-1-[(1*R*,2*S*,5*R*)-(–)-menthoxymethyl]imidazolium bis(trifluoromethylsulfonyl)imides at different temperatures (lines are drawn according to eq 2 in the manuscript, or are straight lines for logarithmic scale).

Figure S4. Dynamic viscosity of chosen 1-alkyl-3-methyl imidazolium ionic liquids, [$C_nC_1$im]$^+$, with anions: tetrafluoroborate, BF_4^- (C_2 – Ref. 34; C_4 – Ref. 35; C_6 – Ref. 36; C_8 – Ref. 14), hexafluorophosphate, PF_6^- (C_4 – Ref. 38; C_6 - Ref. 39; C_8 – Ref. 37), bis(trifluoromethylsulfonyl)imide, NTf_2^- (C_1, C_2 – Ref. 40; C_3 – Ref. 41; C_4 – Ref. 42; C_6 – Ref. 43; C_8 – Ref. 44), symmetrical 1,3-dialkylimidazolium bis(trifluoromethylsulfonyl)-imide [$C_nC_n$im][NTf_2] (Ref. 45) and [C_n-im-CH_2OMen][NTf_2] ILs (n = 1–10) (this work) at T = 298.15 K.

Figure S5. Conductivity at 298.15 K, or given in description, if other. Points: filled triangles: 1-alkyl-1-methylpiperridinium bis(trifluoromethanesulfonyl)imides, [$C_nC_1$pip][NTf_2] at 293.15 K (Ref. 48); filled circles: 1-alkyl-1-methylmorpholinium dicyanamides, [$C_nC_1$mo][DCA] (Ref. 50); empty squares: 1-alkylthiolanium bis(trifluoro-methanesulfonyl)imides, [$C_n$tl][NTf_2] (Ref. 49); empty triangles: 1-alkylthianium bis(trifluoromethanesulfonyl)imides [$C_n$tn][NTf_2] (Ref. 49).

Figure S6. Conductivity of chosen 1-alkyl-3-methyl imidazolium ionic liquids with anions: tetrafluoroborate, BF_4^- (C_2, C_4, C_6 – Ref. 51; C_8 – Ref. 52), hexafluorophosphate, PF_6^- (C_4 – Ref. 53; C_6 – Ref. 54; C_8 – Ref. 52), bis(trifluoromethylsulfonyl)imide, NTf_2^- (C_1 – Ref. 40; C_2 – Ref. 55; C_3 – Ref. 56; C_4 – Ref. 57; C_6, C_8 – Ref. 56) and [C_n-im-CH_2OMen][NTf_2] ILs (n = 1–10) (this work) at T = 298.15 K.

Figure S7. Viscosity at 298.15 K, or given in description, if other. Points: filled diamonds: 1-alkyl-1-methylpyrrolidinium dicyanamides, [$C_nC_1$pyr][DCA] (Ref. 42, 73, 74), filled squares: 1-alkyl-3-methylimidazolium trifluoromethanesulfonates, [$C_nC_1$im][TFO] at 293.15 K (Ref. 34, 75-77); filled triangles: 1-alkyl-1-methylpiperridinium bis(trifluoromethane-sulfonyl)imides, [$C_nC_1$pip][NTf_2] at 293.15 K (Ref. 48); filled circles: 1-alkyl-1-methylmorpholinium dicyanamides, [$C_nC_1$mo][DCA] (Ref. 50); empty diamonds: 1-alkyl-4-methyltriazolium bis(trifluoromethanesulfonyl)imides, [C_nC_1-4-tz][NTf_2] (Ref. 72); empty squares: 1-alkylthiolanium bis(trifluoromethanesulfonyl)imides, [$C_n$tl][NTf_2] (Ref. 49); empty triangles: 1-alkylthianium bis(trifluoromethanesulfonyl)imides [$C_n$tn][NTf_2] (Ref. 49); empty circles: 1-alkylpyridinium bis(trifluoromethanesulfonyl)imides, [$C_n$py][NTf_2] at 353.15 K (Ref.71).

Table S1. Kinematic viscosity, ν ($mm^2 \cdot s^{-1}$) of 1-alkyl-3-[(1*R*,2*S*,5*R*)-(–)-menthoxymetyl]imidazolium bis(trifluoromethylsulfonyl)imide ionic liquids, [C_n-im-CH_2OMen][NTf_2] ILs (n = 3,4,6,9,10) at T = (298.15–328.15) K and p = 0.1 MPa measured with Micro-Ubbelohde viscometers, capillaries IIc and III.

T / K	ν / $mm^2 \cdot s^{-1}$				
	[C_3-im-CH_2OMen][NTf_2]	[C_4-im-CH_2OMen][NTf_2]	[C_6-im-CH_2OMen][NTf_2]	[C_9-im-CH_2OMen][NTf_2]	[C_{10}-im-CH_2OMen][NTf_2]
298.15	675.6	744.6	767.7	724.2	700.3
303.15	429.5	469.0	493.5	476.6	465.4
308.15	283.7	308.5	329.1	324.3	319.1
313.15	194.2	209.5	227	227.5	225.2
318.15	137.4	146.7	161.5	164.2	163.2
323.15	100.1	108.8	118.3	121.6	121.3
328.15	75.06	82.22	89	92.35	92.26

Standard uncertainties: $u(T)$ = 0.05 K, $u(p) \pm 1\times10^{-2}$ MPa, $u(\nu)$ = 0.3%

Table S2. Dynamic viscosity, η (mPa·s) of 1-alkyl-3-[(1*R*,2*S*,5*R*)-(–)-menthoxymetyl]imidazolium bis(trifluoromethylsulfonyl)imide ionic liquids, [C_n-im-CH_2OMen][NTf_2] ILs (n = 1–10) at T = (298.15–328.15) K and p = 0.1 MPa.

T / K	η / mPa · s									
	[C_1-im-CH_2OMen][NTf_2][a]	[C_2-im-CH_2OMen][NTf_2][a]	[C_3-im-CH_2OMen][NTf_2][b]	[C_4-im-CH_2OMen][NTf_2][b]	[C_5-im-CH_2OMen][NTf_2][a]	[C_6-im-CH_2OMen][NTf_2][b]	[C_7-im-CH_2OMen][NTf_2][a]	[C_8-im-CH_2OMen][NTf_2][a]	[C_9-im-CH_2OMen][NTf_2][b]	[C_{10}-im-CH_2OMen][NTf_2][b]
298.15	869.1	745.6	869.8	948.2	1009	948.1	887.2	972.1	869.7	832.6
303.15	536	464.3	551.1	595.1	637.4	607.5	572.3	625.5	570.4	551.4
308.15	345.7	305.3	362.8	390.2	417.1	403.7	382.9	416.4	386.8	376.8
313.15	236.9	207.3	247.5	264.1	284.7	277.5	265.2	287.9	270.4	265.0
318.15	166.8	146.8	174.4	184.3	197.2	196.8	189.8	206.1	194.5	191.4
323.15	121.7	108.1	126.8	136.3	143.2	143.7	139.6	150.7	143.6	141.8
328.15	91.21	81.86	94.71	102.6	106.9	107.7	105.2	113.4	108.7	107.5

[a] Anton Paar DSA 5000M with rolling-ball viscometer Lovis 2000 M; standard uncertainties: $u(T)$ = 0.02 K, $u(p) \pm 1\times10^{-2}$MPa, $u(\eta)$ up to 0.5%; [b] Micro-Ubbelohde viscometers, capillaries IIc and III (calculated from experimental kinematic viscosities and densities reported earlier in Ref. [27]); standard uncertainties: $u(T)$ = 0.05 K, $u(p) \pm 1\times10^{-2}$ MPa,

Table S3. Conductivity, κ (mS·cm^{-1}) of 1-alkyl-3-[(1*R*,2*S*,5*R*)-(–)-menthoxymetyl]imidazolium bis(trifluoromethylsulfonyl)imide ionic liquids, [C_n-im-CH_2OMen][NTf_2] ILs (n = 1–10) at T = (293.15–323.15) K and p = 0.1 MPa.

T / K	κ^a / mS · cm^{-1}									
	[C_1-im-CH_2OMen][NTf_2]	[C_2-im-CH_2OMen][NTf_2]	[C_3-im-CH_2OMen][NTf_2]	[C_4-im-CH_2OMen][NTf_2]	[C_5-im-CH_2OMen][NTf_2]	[C_6-im-CH_2OMen][NTf_2]	[C_7-im-CH_2OMen][NTf_2]	[C_8-im-CH_2OMen][NTf_2]	[C_9-im-CH_2OMen][NTf_2]	[C_{10}-im-CH_2OMen][NTf_2]
293.15	0.0753	0.0922	0.0596	0.0604	0.0499	0.0404	0.0431	0.0405	0.0373	0.0326
298.15	0.1213	0.1466	0.1033	0.0961	0.0778	0.0657	0.0644	0.0657	0.0576	0.0514
303.15	0.1860	0.2225	0.1624	0.1466	0.1174	0.1026	0.0939	0.1026	0.0862	0.0774
308.15	0.2726	0.3233	0.2455	0.2151	0.1713	0.1544	0.1335	0.1544	0.1251	0.1120
313.15	0.3835	0.4513	0.3572	0.3044	0.2424	0.2244	0.1857	0.2244	0.1765	0.1560
318.15	0.5194	0.6074	0.4880	0.4169	0.3335	0.3160	0.2530	0.3160	0.2427	0.2100
323.15	0.6791	0.7902	0.6708	0.5537	0.4470	0.4320	0.3382	0.4321	0.3259	0.2735

^{a} Standard uncertainties: $u(T)$ = 0.02 K, $u(p)$ ± 1×10^{-2} MPa, $u(\kappa)$ = 1.5%

Table S4. Parameters of eq 1 for viscosity (in the manuscript): B_η parameter, limiting viscosity, A_η, Angell's strength parameter, D, with the ideal glass transition temperature, $T_{0\eta}$ = 165.06 K (from the group contribution model).

IL [C_n-im-CH_2OMen][NTf_2]	$A_\eta \cdot 10^2$/ mPa·s	B_η / K	D (= $B_\eta/T_{0\eta}$)
[C_1-im-CH_2OMen][NTf_2]	0.25 ± 0.04	1699 ± 24	10.3
[C_2-im-CH_2OMen][NTf_2]	0.28 ± 0.04	1664 ± 21	10.1
[C_3-im-CH_2OMen][NTf_2]	0.39 ± 0.03	1640 ± 11	9.9
[C_4-im-CH_2OMen][NTf_2]	0.35 ± 0.04	1665 ± 16	10.1
[C_5-im-CH_2OMen][NTf_2]	0.38 ± 0.03	1660 ± 10	10.1
[C_6-im-CH_2OMen][NTf_2]	0.55 ± 0.04	1605 ± 10	9.7
[C_7-im-CH_2OMen][NTf_2]	0.64 ± 0.05	1575 ± 11	9.6
[C_8-im-CH_2OMen][NTf_2]	0.64 ± 0.05	1587 ± 11	9.6
[C_9-im-CH_2OMen][NTf_2]	0.91 ± 0.05	1527 ± 7	9.2
[C_{10}-im-CH_2OMen][NTf_2]	1.08 ± 0.04	1497 ± 5	9.1

Table S5. Parameters of eq 2 for conductivity (in the manuscript): B_κ parameter, limiting conductivity, A_κ, with the ideal glass transition temperature, $T_{0\kappa}$ = 165.06 K (from the group contribution model).

IL [C_n-im-CH_2OMen][NTf_2]	A_κ/ mS·cm^{-1}	- B_κ / K
[C_1-im-CH_2OMen][NTf_2]	5224 ± 922	1413 ± 27
[C_2-im-CH_2OMen][NTf_2]	5063 ± 850	1384 ± 26
[C_3-im-CH_2OMen][NTf_2]	11648 ± 1816	1543 ± 24
[C_4-im-CH_2OMen][NTf_2]	5322 ± 615	1449 ± 18
[C_5-im-CH_2OMen][NTf_2]	4536 ± 272	1458 ± 9
[C_6-im-CH_2OMen][NTf_2]	8631 ± 699	1565 ± 12
[C_7-im-CH_2OMen][NTf_2]	2342 ± 34	1398 ± 2
[C_8-im-CH_2OMen][NTf_2]	8638 ± 686	1565 ± 12
[C_9-im-CH_2OMen][NTf_2]	3156 ± 111	1450 ± 5
[C_{10}-im-CH_2OMen][NTf_2]	1689 ± 250	1378 ± 23

Table S6. Molar conductivity, Λ (S·cm^2·mol^{-1}) of 1-alkyl-3-[(1*R*,2*S*,5*R*)-(–)-menthoxymetyl]imidazolium bis(trifluoromethylsulfonyl)imide ionic liquids, [C_n-im-CH_2OMen][NTf_2] ILs (n = 1–10) at T = (298.15–323.15) K and p = 0.1 MPa.

T / K	Λ / S · cm^2·mol^{-1}									
	[C_1-im-CH_2OMen][NTf_2]	[C_2-im-CH_2OMen][NTf_2]	[C_3-im-CH_2OMen][NTf_2]	[C_4-im-CH_2OMen][NTf_2]	[C_5-im-CH_2OMen][NTf_2]	[C_6-im-CH_2OMen][NTf_2]	[C_7-im-CH_2OMen][NTf_2]	[C_8-im-CH_2OMen][NTf_2]	[C_9-im-CH_2OMen][NTf_2]	[C_{10}-im-CH_2OMen][NTf_2]
298.15	0.048	0.061	0.046	0.058	0.036	0.032	0.032	0.029	0.029	0.026
303.15	0.073	0.093	0.072	0.087	0.054	0.050	0.047	0.044	0.043	0.039
308.15	0.108	0.135	0.108	0.125	0.079	0.076	0.068	0.064	0.063	0.057
313.15	0.154	0.190	0.155	0.174	0.112	0.110	0.094	0.091	0.089	0.080
318.15	0.210	0.256	0.215	0.235	0.154	0.156	0.129	0.127	0.123	0.108
323.15	0.271	0.335	0.289	0.309	0.207	0.214	0.173	0.175	0.166	0.141

Table S7. Linear fitting parameters for fractional Walden plots according to linear form of eq 3 (in the manuscript) and ionicity expressed as a distance from ideal Walden Line

IL [C_n-im-CH_2OMen][NTf_2]	α	log (*const/* S · cm^2·mol^{-1})	ionicity
[C_1-im-CH_2OMen][NTf_2]	-0.4840 ± 0.0043	0.8918 ± 0.0076	0.33
[C_2-im-CH_2OMen][NTf_2]	-0.4440 ± 0.0015	0.8809 ± 0.0029	0.36
[C_3-im-CH_2OMen][NTf_2]	-0.4361 ± 0.0033	0.9551 ± 0.0058	0.37
[C_4-im-CH_2OMen][NTf_2]	-0.3978 ± 0.0020	0.8577 ± 0.0033	0.4
[C_5-im-CH_2OMen][NTf_2]	-0.5434 ± 0.0014	0.9027 ± 0.0022	0.29
[C_6-im-CH_2OMen][NTf_2]	-0.51078 ± 0.00018	1.0067 ± 0.0003	0.31
[C_7-im-CH_2OMen][NTf_2]	-0.6370 ± 0.0049	0.9052 ± 0.0083	0.23
[C_8-im-CH_2OMen][NTf_2]	-0.5930 ± 0.0064	0.962 ± 0.010	0.26
[C_9-im-CH_2OMen][NTf_2]	-0.6282 ± 0.0011	0.9715 ± 0.0018	0.24
[C_{10}-im-CH_2OMen][NTf_2]	-0.6995 ± 0.0057	0.9546 ± 0.0099	0.2

Table S8. *RD* values obtained from eq 7 (in the manuscript) for each IL in temperature range *T* = 298.15 K-328.15 K.

IL [C_n-im-CH_2OMen][NTf_2]	Percent deviation (range) (%)	Mean percent deviation (%)
[C_1-im-CH_2OMen][NTf_2]	-0.8; 6.9	1.7
[C_2-im-CH_2OMen][NTf_2]	-16.3; -8.8	13.1
[C_3-im-CH_2OMen][NTf_2]	1.7; 5.3	3.1
[C_4-im-CH_2OMen][NTf_2]	6.2; 11.9	8.9
[C_5-im-CH_2OMen][NTf_2]	11.4; 16.3	13.5
[C_6-im-CH_2OMen][NTf_2]	9.1; 14.1	10.6
[C_7-im-CH_2OMen][NTf_2]	2.7; 10.9	5.5
[C_8-im-CH_2OMen][NTf_2]	10.1; 16.3	12.1
[C_9-im-CH_2OMen][NTf_2]	-1.2; 12.3	4.7
[C_{10}-im-CH_2OMen][NTf_2]	-6.9; 9.8	1.3

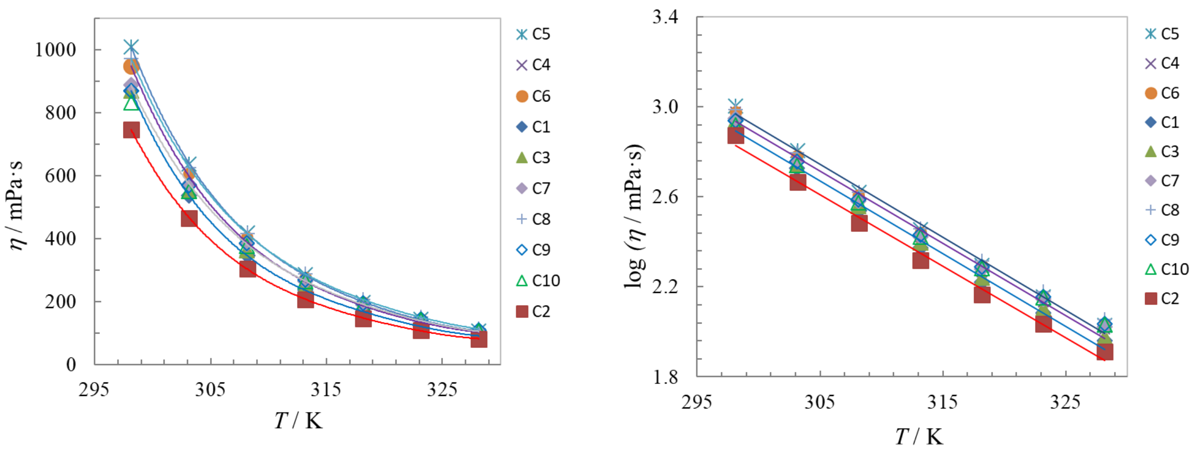

Figure S1. Dynamic viscosity, η, (left) and log η (right) of 3-alkyl-1-[(1*R*,2*S*,5*R*)-(–)-menthoxymethyl]imidazolium bis(trifluoromethylsulfonyl)imides at different temperatures (for better visibility only chosen lines are drawn according to eq 1 in the manuscript, or are straight lines for logarithmic scale).

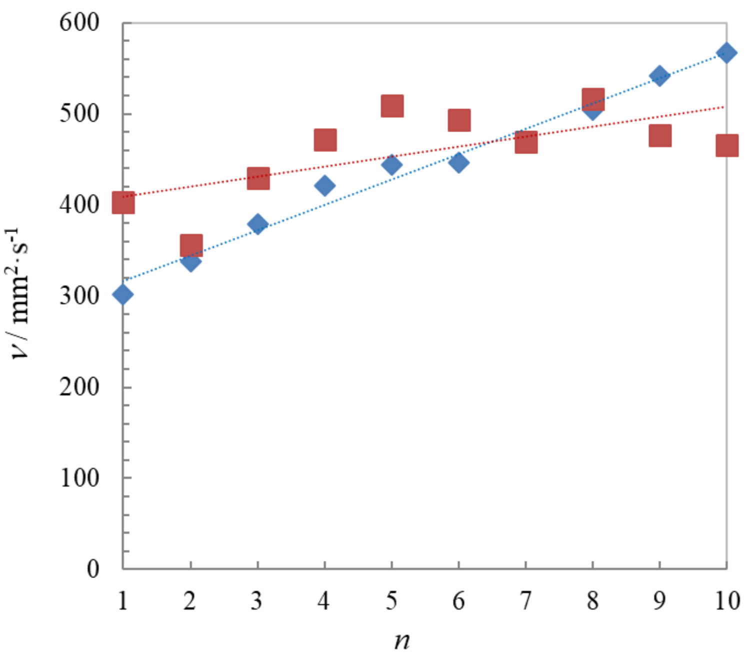

Figure S2. Kinematic viscosity of [C_n-im-CH_2OMen][NTf_2] ILs *vs.* *n* (*n* = 1–10) at *T* = 303.15 K obtained in this work (squares) in comparison with literature values (diamonds) (Ref. 25).

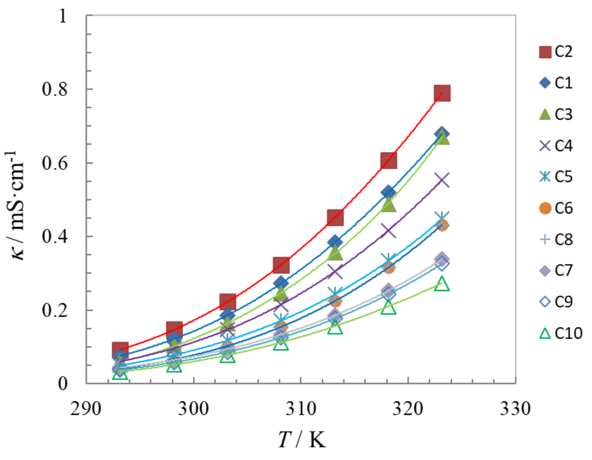

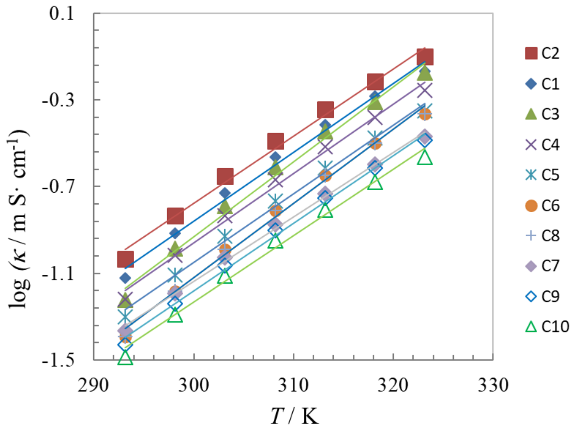

Figure S3. Conductivity, κ, (left) and log κ (right) of 3-alkyl-1-[(1*R*,2*S*,5*R*)-(–)-menthoxymethyl]imidazolium bis(trifluoromethylsulfonyl)imides at different temperatures (lines are drawn according to eq 2 in the manuscript, or are straight lines for logarithmic scale).

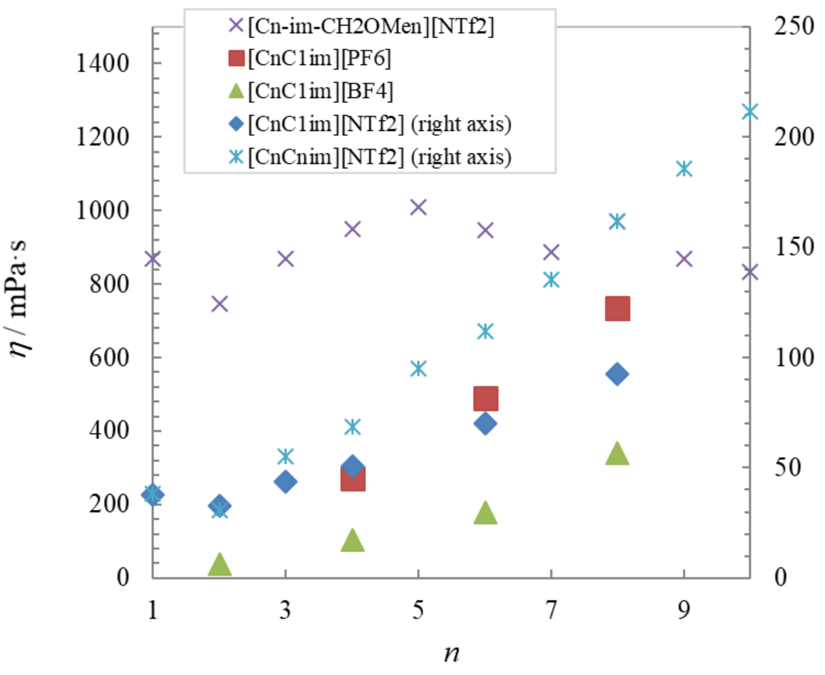

Figure S4. Dynamic viscosity of chosen 1-alkyl-3-methyl imidazolium ionic liquids, $[C_nC_1im]^+$, with anions: tetrafluoroborate, BF_4^- (C_2 – Ref. 34; C_4 – Ref. 35; C_6 – Ref. 36; C_8 – Ref. 14), hexafluorophosphate, PF_6^- (C_4 – Ref. 38; C_6 - Ref. 39; C_8 – Ref. 37), bis(trifluoro-methylsulfonyl)imide, NTf_2^- (C_1, C_2 – Ref. 40; C_3 – Ref. 41; C_4 – Ref. 42; C_6 – Ref. 43; C_8 – Ref. 44), symmetrical 1,3-dialkylimidazolium bis(trifluoromethylsulfonyl)imide $[C_nC_nim][NTf_2]$ (Ref. 45) and $[C_n\text{-im-}CH_2OMen][NTf_2]$ ILs (n = 1–10) (this work) at T = 298.15 K.

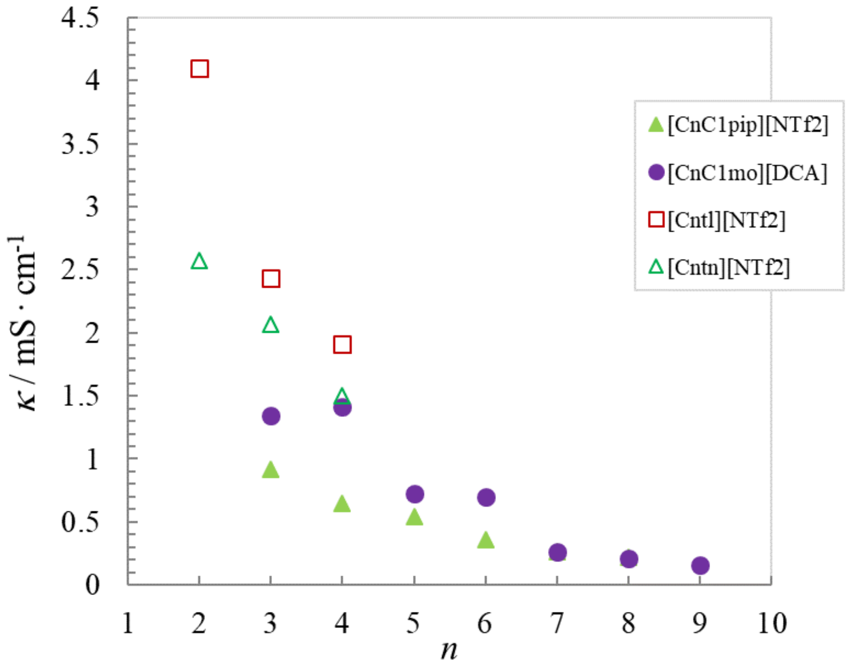

Figure S5. Conductivity at 298.15 K, or given in description, if other. Points: filled triangles: 1-alkyl-1-methylpiperridinium bis(trifluoromethanesulfonyl)imides, [$C_nC_1$pip][NTf_2] at 293.15 K (Ref. 48); filled circles: 1-alkyl-1-methylmorpholinium dicyanamides, [$C_nC_1$mo][DCA] (Ref. 50); empty squares: 1-alkylthiolanium bis(trifluoromethane-sulfonyl)imides, [$C_n$tl][NTf_2] (Ref. 49); empty triangles: 1-alkylthianium bis(trifluoromethanesulfonyl)imides [$C_n$tn][NTf_2] (Ref. 49).

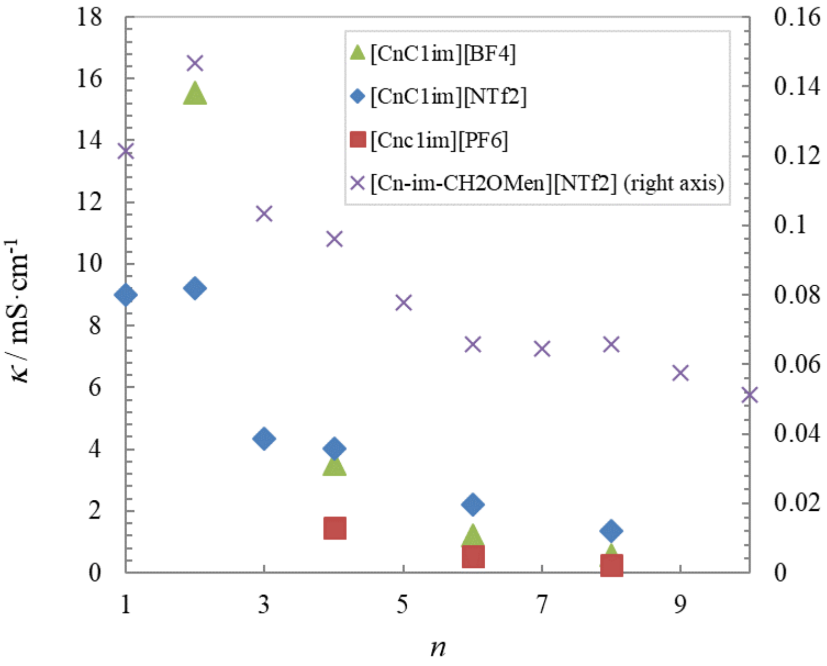

Figure S6. Conductivity of chosen 1-alkyl-3-methyl imidazolium ionic liquids with anions: tetrafluoroborate, BF_4^- (C_2, C_4, C_6 – Ref. 51; C_8 – Ref. 52), hexafluorophosphate, PF_6^- (C_4 – Ref. 53; C_6 – Ref. 54; C_8 – Ref. 52), bis(trifluoromethylsulfonyl)imide, NTf_2^- (C_1 – Ref. 40; C_2 – Ref. 55; C_3 – Ref. 56; C_4 – Ref. 57; C_6, C_8 – Ref. 56) and [C_n-im-CH_2OMen][NTf_2] ILs (n = 1–10) (this work) at T = 298.15 K.

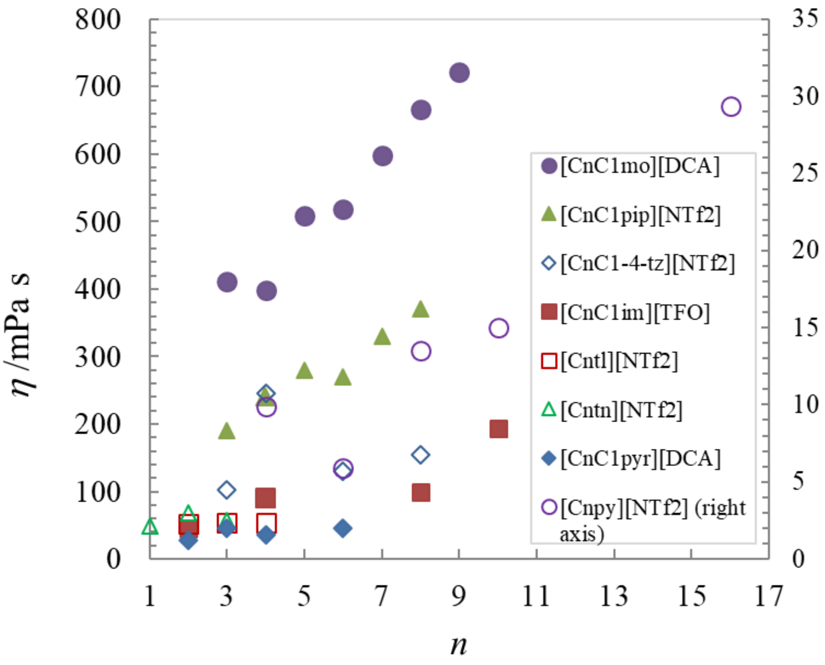

Figure S7. Viscosity at 298.15 K, or given in description, if other. Points: filled diamonds: 1-alkyl-1-methylpyrrolidinium dicyanamides, [$C_nC_1$pyr][DCA] (Ref. 42, 73, 74), filled squares: 1-alkyl-3-methylimidazolium trifluoromethanesulfonates, [$C_nC_1$im][TFO] at 293.15 K (Ref. 34, 75-77); filled triangles: 1-alkyl-1-methylpiperridinium bis(trifluoromethane-sulfonyl)imides, [$C_nC_1$pip][NTf_2] at 293.15 K (Ref. 48); filled circles: 1-alkyl-1-methylmorpholinium dicyanamides, [$C_nC_1$mo][DCA] (Ref. 50); empty diamonds: 1-alkyl-4-methyltriazolium bis(trifluoromethanesulfonyl)imides, [C_nC_1-4-tz][NTf_2] (Ref. 72); empty squares: 1-alkylthiolanium bis(trifluoromethanesulfonyl)imides, [$C_n$tl][NTf_2] (Ref. 49); empty triangles: 1-alkylthianium bis(trifluoromethanesulfonyl)imides [$C_n$tn][NTf_2] (Ref. 49); empty circles: 1-alkylpyridinium bis(trifluoromethanesulfonyl)imides, [$C_n$py][NTf_2] at 353.15 K (Ref.71).
